# Gesundheitsfördernde Arbeitsgestaltung im Homeoffice im Kontext der COVID-19-Pandemie

**DOI:** 10.1007/s40664-020-00419-1

**Published:** 2021-01-07

**Authors:** Natascha Mojtahedzadeh, Elisabeth Rohwer, Julia Lengen, Volker Harth, Stefanie Mache

**Affiliations:** Zentralinstitut für Arbeitsmedizin und Maritime Medizin (ZfAM), Universitätsklinikum Hamburg-Eppendorf (UKE), Seewartenstraße 10 | Haus 1, 20459 Hamburg, Deutschland

**Keywords:** Corona, Heimarbeitsplatz, Arbeitsplatzgestaltung, Ergonomie, Regeneration, Gesundheitsförderung, Corona, Working from home, Workstation design, Ergonomics, Recovery, Health promotion

## Abstract

**Hintergrund:**

Die mit der COVID-19-Pandemie einhergehenden Kontaktbeschränkungen haben viele Unternehmen dazu veranlasst, ihren Beschäftigten aus Gründen des Infektionsschutzes das Arbeiten aus dem Homeoffice zu ermöglichen.

**Fragestellung:**

In dieser Literaturübersicht wird der Frage nachgegangen, inwiefern die Ausübung der beruflichen Tätigkeit im Homeoffice gesundheitsfördernd gestaltet werden kann.

**Ergebnisse:**

Arbeitsplätzen im Homeoffice liegen dieselben Richtlinien wie für Büro- und Bildschirmarbeitsplätze zugrunde. Um negative psychische und physische Beanspruchungsfolgen zu vermeiden, wird die ergonomische Arbeitsplatzgestaltung empfohlen. Außerdem kommt der Arbeitszeitgestaltung (Strukturierung des Arbeitstages, Einhalten von Pausen und Regenerationseinheiten und die Vermeidung von Störungen und Unterbrechungen) eine hohe Bedeutung für eine gesundheitsfördernde Arbeitsweise im Homeoffice zu.

**Schlussfolgerung:**

Wichtige Bausteine für gesundheitsfördernde Arbeitsgestaltung sind die zeitliche, räumliche und insbesondere mentale Trennung von Arbeits- und Privatleben. Bei der gesundheitsfördernden Arbeitsplatzgestaltung im Homeoffice sind Beschäftigte und ihre individuellen Bedürfnisse zu berücksichtigen. Die Verantwortung der Realisierung gesundheitsfördernder Arbeitsgestaltung liegt durch die ad-hoc-Umstellung in der Pandemie jedoch zumeist allein bei den Beschäftigten.

## Abstract (en)

**Background:**

The contact restrictions associated with the COVID-19 pandemic have led many companies to allow their employees to work from home for infection control reasons.

**Objective:**

This literature review explores the question in what way health-promoting work from home can be excercised.

**Results:**

Working from home requires consideration of the same guidelines just like those for office and computer workstations. To prevent negative mental and physical stress reactions, an ergonomic workstation design is recommended. Furthermore, the organization of working time (structuring the working day, adhering to breaks and regeneration units, and avoiding disturbances and interruptions) is of great importance for a health-promoting work design at home.

**Conclusion:**

Important components of a health-promoting work design are temporal, spatial and mental separation of work and private life. Employees and their individual needs must be taken into account when designing a health-promoting workplace at home; however, the responsibility for implementing health-promoting work design mostly lies solely with the employees due to the ad hoc change of workplace.

Die COVID-19-Pandemie („coronavirus disease“ 2019, deutsch: Coronavirus-Krankheit-2019) führte dazu, im Sinne des Infektionsschutzes, viele Beschäftigte kurzfristig ins Homeoffice zu entsenden [27]. Im Kontext flexibler Arbeitsformen wie der Homeoffice-Tätigkeit hat sich in Studien bereits gezeigt, dass die Überschneidung von Arbeits- und Wohnort der Beschäftigten mit einigen Ressourcen, aber auch verschiedenen psychischen Belastungsfaktoren einhergehen kann [6]. Die plötzliche Neuorganisation des Arbeitsplatzes sowie die andauernde Situation der COVID-19-Pandemie können auch mit einem erhöhten Stresserleben verbunden sein [29, 50]. Daher ist eine gesundheitsfördernde Arbeitsgestaltung im Homeoffice notwendig.

Basierend auf dem Beschluss der Bundesregierung vom 15. April 2020 „Beschränkungen des öffentlichen Lebens zur Eindämmung der COVID-19-Epidemie“ [11] haben viele Arbeitgeber die Büroarbeitsplätze ihrer Beschäftigten kurzfristig ins Homeoffice verlegt [27]. Das Arbeiten im Homeoffice kann in Form eines Telearbeitsplatzes nach § 2 Abs. 7 der Verordnung über Arbeitsstätten (Arbeitsstättenverordnung – ArbStättV; [57]) oder des mobilen Arbeitens umgesetzt werden. Während dem Telearbeitsplatz eine vertragliche Vereinbarung zwischen Arbeitgeber und Beschäftigtem zugrunde liegt, ist der mobile Arbeitsplatz in Deutschland bisher noch nicht legal definiert [17, 57]. Laut der ArbStättV sind Telearbeitsplätze „fest eingerichtete Bildschirmarbeitsplätze im Privatbereich der Beschäftigten“ mit vertraglich vereinbarter wöchentlicher Arbeitszeit [57]. Mobiles Arbeiten hingegen beschreibt eine ortsunabhängige Arbeitstätigkeit von mindestens zehn Wochenstunden, die nicht an einen festen Arbeitsplatz, wie z. B. das Zuhause, gebunden ist [22]. Durch die ad hoc eingetretene Homeoffice-Situation für viele Beschäftigte liegt es nahe, dass in kurzer Zeit keine vertraglichen Vereinbarungen getroffen werden konnten. Daher ist davon auszugehen, dass der Großteil der Beschäftigten, die durch die COVID-19-Pandemie bedingt im Homeoffice tätig sind, in einer Mischform von Telearbeit und mobiler Arbeit ihre Arbeit von zu Hause verrichtet: Der Arbeitsplatz wird (entsprechend der Telearbeit) ortsgebunden in die Wohnung der Beschäftigten verlegt, jedoch ohne vertragliche Vereinbarung und damit einhergehende gesetzliche Verpflichtungen (vgl. mobiles Arbeiten). Im Folgenden wird diese besondere Form als Homeoffice-Tätigkeit angenommen.

Die Einrichtung eines ergonomischen Arbeitsplatzes im Homeoffice stellt indes eine maßgebende Herausforderung unter den aktuellen Umständen der COVID-19-Pandemie dar.

Neue Arbeitssituation im Homeoffice unterliegt besonderem Augenmerk

Wissenschaftliche Ergebnisse deuten darauf hin, dass die Überlagerung von Berufs- und Privatleben von verschiedenen psychischen Belastungsfaktoren und Ressourcen gekennzeichnet ist [6]. Häufig verändern sich in flexiblen Arbeitsbedingungen wie der Homeoffice-Tätigkeit die Handlungsspielräume der Beschäftigten dahingehend, dass ihnen zwangsweise mehr Verantwortung in Bezug auf die Arbeitsgestaltung und die eigene Gesundheit übertragen wird [60, 64]. Sie gestalten zwangsläufig viele Aspekte ihres Arbeitsplatzes weitgehend eigenständig. Die damit einhergehende Autonomie und Flexibilität kann sich für die Beschäftigten sowohl gesundheitsfördernd als auch gesundheitsbelastend auswirken [14]. Neben der flexiblen und eigenverantwortlichen Arbeitszeitgestaltung ist auch die Einhaltung und Gestaltung von Regenerationseinheiten in Form von Ruhepausen und -zeiten für eine gesundheitsfördernde Arbeitsgestaltung im Homeoffice entscheidend.

## Methodik

Vor dem Hintergrund dieser Herausforderungen behandelt dieser Übersichtsartikel die gesundheitsfördernde Arbeitsgestaltung im Homeoffice in Zeiten der COVID-19-Pandemie unter besonderer Berücksichtigung der Themenfelder (ergonomische) Arbeitsplatzgestaltung, Arbeitszeitgestaltung und Regeneration. Zugrunde liegen in erster Linie wissenschaftliche Studien, die die gesundheitsfördernde Arbeitsgestaltung im Homeoffice fokussieren, anhand einer explorativen Literaturrecherche in wissenschaftlichen Datenbanken, u. a. PubMed und PsychINFO, recherchiert. Außerdem wird sich auf die geltenden gesetzlichen Normen und Verordnungen gestützt. Unter Arbeitsgestaltung wird in diesem Übersichtsartikel die Gestaltung von ergonomischen Arbeitsplätzen, Arbeits- und Erholungszeit gefasst. Nach der Beschreibung möglicher Herausforderungen werden praktische Umsetzungsempfehlungen für Arbeitgeber und Beschäftigte formuliert.

## Ergonomische Arbeitsplatzgestaltung im Homeoffice

Um psychische und physische Beanspruchungsfolgen zu vermeiden, muss die Arbeitsplatzgestaltung an die individuellen Bedürfnisse der Beschäftigten angepasst werden. Zur Reduzierung möglicher negativer Beanspruchungsfolgen können die Belastungen in ihrer Ausprägung u. a. durch technische (ergonomische), organisatorische und personenbezogene Maßnahmen für die Neu- bzw. Umgestaltung des Arbeitsplatzes im Homeoffice vermindert werden (TOP-Prinzip nach § 4 Arbeitsschutzgesetz, ArbSchG; [18, 25, 54]). In diesem Artikel werden technische Maßnahmen mit Hinblick auf den Fokus der Arbeitsorganisation insbesondere in Form von Ergonomie berücksichtigt, um hier vorliegend einen übergeordneten Schwerpunkt zu legen. Eine Grundpflicht des Arbeitgebers besteht in der Vermeidung von Gefährdungen für die Beschäftigten (§ 4 der Verordnung über Sicherheit und Gesundheitsschutz bei der Verwendung von Arbeitsmitteln Betriebssicherheitsverordnung (BetrSichV) [58]). Zur Erfüllung dieser Pflicht, aber auch zur Förderung von Leistung und Produktivität der Beschäftigten, sollten ergonomische Arbeitsmittel bereitgestellt werden [28, 54]. Grundsätzlich gelten für die ergonomische Gestaltung von Homeoffice-Arbeitsplätzen die gleichen Vorgaben wie für Büro- und Bildschirmarbeitsplätze [59]. Diese sind in Tab. 1 dargestellt.

**Table 1:** 

Beleuchtung	Ausreichende Lichtverhältnisse (500 lx, tageslichtdurchlässig), für Beschäftigte ab 60 Jahre werden bis zu 1000 lx empfohlen
Raumtemperatur	Für sitzende Körperhaltungen in Arbeitsräumen mindestens 20 °C, für stehende oder schwerere Arbeitstätigkeiten sowie höhere Außentemperaturen (über 26 °C) Maßnahmen wie z. B. Lüften und Verminderung der Sonneneinstrahlung ergreifen
Lärm	Hintergrundgeräuschlose, konzentrationsfördernde Arbeitsumgebung [maximal 45–55 dB(A)]
Bildschirm und Eingabemittel	Abstand von Person zu Bildschirm sollte 50–70 cm betragen Störende Reflexionen und Blendungen sind zu vermeiden Bildschirm und Eingabemittel (z. B. Maus, Tastatur) sollten flexibel und ergonomisch angeordnet werden können Eingabemittel sollten getrennt vom Bildschirm sein (z. B. externe Tastatur und Maus statt Nutzung von Tastatur und Touchpad eines Laptops)

Darüber hinaus können regelmäßige Blicke in die Ferne die Augen bei längerer Bildschirmtätigkeit entspannen [52]. Ein Wechsel mit Arbeitsphasen im Stehen sowie regelmäßiges Bewegen der Gliedmaßen stellen gesundheitsfördernde Maßnahmen für die Schreibtischtätigkeit dar [30, 33, 59]. Die ergonomische Bildschirmtätigkeit im Homeoffice beinhaltet auch die Verwendung von Zubehör wie Tastatur, Maus, Headset und externen Bildschirmen, die eine gesundheitsfördernde Körperhaltung während der Arbeit ermöglichen. Dieses Zubehör erfordert jedoch auch eine entsprechende Arbeitsfläche (optimal: höhenverstellbarer Schreibtisch von 160 × 80 cm; [16]).

Die kurzfristige Umstellung der Arbeitsausführung in das Homeoffice während der COVID-19-Pandemie kann vielen Beschäftigten die Umsetzung ergonomischer Arbeitsplatzgestaltung erschweren [27, 30]. Insbesondere können finanzielle Mittel für die Beschaffung ergonomischer Möbel in Bezug auf das Arbeiten von zu Hause fehlen [30]. Anders als bei einem vertraglich vereinbarten Telearbeitsplatz, welcher dieses bereits voraussetzt [57], liegt die Verantwortung in der aktuellen Situation allein bei den Beschäftigten. Eine qualitative Studie von Janneck et al. zeigt, dass die Mehrheit der im Homeoffice Tätigen zwar laut eigenen Angaben effektiv arbeiten kann, jedoch nur drei von 41 Befragten über ergonomische Arbeitsmittel zu Hause verfügen. Rund ein Drittel versucht die ergonomischen Empfehlungen zu befolgen, beispielsweise durch die Nutzung ergonomischer Stühle und höhenverstellbarer Tische [30]. Letztlich bleibt die Arbeitsplatzgestaltung von den individuellen Rahmenbedingungen und Bedürfnissen der Beschäftigten im Homeoffice abhängig. Eine gesundheitsfördernde Arbeitsplatzgestaltung liegt damit in der Verantwortung der Beschäftigten.

## Flexible Arbeitszeitgestaltung im Homeoffice

Die im Homeoffice vorliegenden flexiblen und selbst zu gestaltenden Arbeitsbedingungen erfordern insbesondere Selbstmanagement- und Arbeitsgestaltungskompetenzen, wie z. B. Selbstführung und die Eigenverantwortung über die Strukturierung von Arbeitsaufgaben sowie Motivierungsstrategien [14]. Eine damit einhergehende eigenverantwortliche Arbeitszeitgestaltung stellt einen höheren Anspruch an die Selbstorganisation der Beschäftigten (im Homeoffice) dar [35]. Als unterstützende Methoden zur Arbeitszeitplanung können beispielsweise einfache To-do-Listen [12], die ALPEN-Methode (Aufgaben notieren, Länge einschätzen, Pufferzeiten einplanen, Entscheidungen treffen und Nachkontrolle vornehmen [39]) dienen oder das Eisenhower-Prinzip der Einteilung und Priorisierung von Aufgaben nach Dringlichkeit und Wichtigkeit [21] genutzt werden. Darüber hinaus deuten wissenschaftliche Analysen darauf hin, dass die Arbeitsproduktivität auch durch die Setzung von Zielen und eine kontrollierte Zeitplanung verstärkt werden kann [36, 45]. In diesem Zuge werden Monats- oder Jahresziele relevant. Präzise formulierte (Arbeits‑)Ziele können die Leistungsfähigkeit nachweislich steigern [34]. Hierbei kann z. B. das SMART-Prinzip zur Zielformulierung (spezifisch, messbar, akzeptiert [z. B. von Teammitgliedern], realistisch und terminiert [20]) unterstützend sein.

Unfreiwillige Arbeitsunterbrechungen können die Arbeitsproduktivität hingegen vermindern [10] und ebenso wie die gleichzeitige Bearbeitung mehrerer Aufgaben einen arbeitsbezogenen Belastungsfaktor darstellen [5, 38]. Insbesondere werden Arbeitsstörungen und Unterbrechungen mit einem erhöhten Stresserleben [10, 32], höheren Fehlerquoten [10] und einer geringeren Arbeitsproduktivität [51] assoziiert. Bisherige Forschungsergebnisse zeigen, dass Beschäftige im Homeoffice im Vergleich zur Tätigkeit am Büroarbeitsplatz in ihrer Arbeit weniger gestört werden [4]. Durch die räumliche Distanz zu Kolleg*innen und Führungskräften und damit physischer Abwesenheit muss vermehrt auf digitale Kommunikation zurückgegriffen werden. E‑Mail-Verkehr, Audio- und Videogespräche oder Chatnachrichten rücken für die notwendige Zusammenarbeit in den Fokus und werden somit verstärkt genutzt [23]. Der Einsatz digitaler Arbeitsmittel, beispielweise durch eingehende E‑Mails [9], birgt dabei ein erhöhtes Risiko für Unterbrechungen und Informationsflut [31, 42, 53].

Die Umstände der COVID-19-Pandemie bergen darüber hinaus weitere Herausforderungen in der Arbeitszeitgestaltung, denen sich insbesondere Eltern stellen mussten. Da Schulen und Kindertagesstätten zeitweise geschlossen waren, mussten im Homeoffice tätige Eltern ggf. zusätzlich ihre Kinder zu Hause betreuen und versorgen [27]. Auch für Paare oder Wohngemeinschaften, in denen sich mehrere Mitbewohner*innen gleichzeitig zu Hause aufhalten, können zusätzliche Belastungen durch unkontrollierbare Unterbrechungen entstehen. Derartige beeinträchtigende Arbeitsunterbrechungen sollten nach Möglichkeit unterbunden werden, da diese das Stresserleben begünstigen können [46, 47]. Beschäftigten im Homeoffice wird angeraten, sich feste Zeiten zum Arbeiten einzuteilen, um eine klare Abgrenzung von Arbeits- und Freizeit zu erzielen und Störungen und Unterbrechungen zu reduzieren [24].

## Ruhepausen und -zeiten im Homeoffice

Die Gestaltung von Ruhepausen und -zeiten (Pausen während und Regeneration nach der Arbeit) obliegt durch die besondere Arbeitssituation im Homeoffice einem besonderen Augenmerk. Die eigenverantwortliche Arbeitszeitgestaltung im Homeoffice birgt die Gefahr, dass über die regulären Zeiten hinaus gearbeitet wird. Dies kann für Beschäftigte im Homeoffice psychisch belastend sein [35]. Im Falle einer Erkrankung, z. B. Erkältung o. Ä., könnte durch die Unsichtbarkeit im Homeoffice die Hemmschwelle gegenüber Kolleg*innen und Führungskräften erhöht sein und damit Präsentismus (Ausübung der Arbeitstätigkeit trotz Erkrankung) begünstigen. Daher stellt die Beachtung der Präsentismus-Thematik einen besonders relevanten und sensiblen Sachverhalt für Homeoffice-Tätige dar [37]. Für Führungskräfte und Kolleg*innen ist dieses Verhalten insbesondere im Homeoffice nicht direkt ersichtlich. Daraus resultiert auch die große Relevanz von regelmäßigen Regenerationseinheiten für im Homeoffice Tätige [7].

Die Realisierung einer gesundheitsfördernden Pausenorganisation obliegt laut § 3 ArbSchG als eine Grundpflicht dem Arbeitgeber. Gemäß § 15 des ArbSchG sind Beschäftigte entsprechend dazu verpflichtet, die gesetzlich verpflichtenden Pausenzeiten (§ 4 Arbeitszeitgesetz, ArbZG) einzuhalten [1]. Demnach sollen nach spätestens sechs Stunden verrichteter Arbeit 30 Minuten Pause eingehalten werden. Ein mehr als neunstündiger Arbeitstag sollte von 45 Minuten Pause unterbrochen sein. Überdies müssen Beschäftigte nach Beendigung der täglichen Arbeitszeit mindestens eine elfstündige Ruhezeit einhalten (§ 5 ArbZG [1]). Die Einhaltung von Arbeits- und Erholungszeiten mag für Beschäftigte jedoch aufgrund der flexibleren Arbeitszeiteinteilung im Homeoffice erschwert sein [27]. Ein potenzieller Konflikt durch die Diffusion von Arbeits- und Privatleben kann durch den gemeinsamen Arbeits- und Freizeitort noch verstärkt werden [43]. Flexible Arbeitszeiten können Arbeitszeitmuster verschieben und zu asynchronen Arbeitszeiten führen. Folglich kann ein erhöhter Abstimmungs- und Koordinationsbedarf entstehen, der eine erweiterte Erreichbarkeit mit sich bringen kann [40]. Mit der arbeitsbezogenen Erreichbarkeit und der damit einhergehenden Durchlässigkeit der Grenzen von Arbeits- und Privatleben werden negative Effekte auf das Wohlbefinden der Beschäftigten und Beeinträchtigungen ihres Privatlebens assoziiert [48]. Nachteilig für die Einhaltung der Pausen im Homeoffice könnte die Abwesenheit von Kolleg*innen und/oder der Führungskraft sein, welche möglicherweise durch Verabredungen ansonsten zu (gemeinsamen) Pausen anregen [13]. Um negative Beanspruchungsfolgen wie psychischer Erschöpfung vorzubeugen, sollten dennoch die gesetzlichen Mindestarbeits- und -ruhezeiten eingehalten werden [1, 18, 62].

Homeoffice-Tätigkeit erfordert Selbstmanagementkompetenzen

Hierfür können unterstützende Methoden wie die Pomodoro-Technik [12] angewandt werden, indem ein Wecker oder digitale Tools [49] an vorab festgelegte Arbeits- und Pausenzeiten erinnern. Die Vorhersehbarkeit von Pausen und das gesicherte Pausenzeitfenster unterstützen den Erholungseffekt [62]. Insbesondere bei wahrgenommener Ermüdung sollten festgelegte Pausenzeiten und flexible, bedarfsentsprechende Kurzpausen kombiniert werden [56]. Da der Erholungsgewinn mit zunehmender Länge der Pausen sinkt [61], sollten eher eine Langpause (z. B. Mittagspause) und mehrere Kurzpausen von 5‑ bis 15-minütiger Dauer eingelegt werden [62]. Arbeitstätigkeit und individuelle Bedürfnisse der Beschäftigten bestimmen Anzahl, Dauer und inhaltliche Gestaltung der Pause. Pausen sollten daher möglichst arbeitstätigkeitskompensatorisch gestaltet werden [62]. Bei geringer Aufgabenvielfalt werden mehrere Kurzpausen, bei geringem sozialem Austausch oder Kooperationsanforderungen aktive Pausen mit digitalem Austausch z. B. mit Kolleg*innen empfohlen [8, 61]. Bei repetitiven Tätigkeiten werden stündliche Kurzpausen angeraten, bei kognitiv anspruchsvollen Tätigkeiten alle zwei Stunden [61].

Neben der physischen Distanzierung von der Arbeit, welche im Homeoffice maßgeblich erschwert sein kann, ist hierbei auch das mentale Abschalten von der Arbeit (sog. Detachment) von großer Relevanz. Arbeitsbezogene Belastungsfaktoren können mit geringem Detachment kombiniert zur Entstehung negativer Beanspruchungsfolgen beitragen. Studienergebnisse zeigen, dass Detachment psychische Arbeitsbelastungswirkungen und möglichen negativen Beanspruchungsfolgen (wie beispielsweise Ermüdung) abpuffern kann [63]. Durch die räumliche Nähe von Arbeits- und Privatleben im Homeoffice können ein festgelegter Arbeitsort in der Wohnung und ein bewusster Ortswechsel in Pausen- und Erholungszeiten Detachment unterstützen [44]. Überdies können unabgeschlossene Aufgaben Möglichkeiten der Erholung erschweren. Dies wird begünstigt durch die Option, den Laptop jederzeit wieder aufklappen zu können [55]. In diesem Fall können Rituale zu Beginn und Abschluss der Arbeitszeit das Detachment fördern (z. B. Dokumentation der Arbeitszeit, bewusstes Zuklappen und Verstauen des Dienstlaptops). Auf diese Weise kann der Entstehung negativer gesundheitlicher Konsequenzen wie Erschöpfung und Schlafproblemen vorgebeugt werden [41]. Durch das Verschwimmen der Grenzen von Arbeits- und Privatleben im Homeoffice ist zudem die Gefahr einer Entgrenzung erhöht, welche in geleisteten Überstunden und Unterschreitung der Mindestruhezeiten von elf Stunden münden kann [4]. Verkürzte Ruhezeiten und tägliche Überstunden können sich außerdem negativ auf die Work-Life-Balance auswirken und psychosomatische Beschwerden zur Folge haben [3].

## Umsetzungsimplikationen einer gesundheitsfördernden Arbeitsgestaltung im Homeoffice

Grundsätzlich sollten Arbeitgeber in der aktuellen Situation eine besonders beratende Funktion für Beschäftigte in Bezug auf ergonomische Arbeitsplatzgestaltung im Homeoffice einnehmen. Während der Tätigkeit im Homeoffice kann eine wechselnde Körperhaltung (sitzend/stehend) hilfreich sein [33]. Die Verwendung möglichst ergonomischer Arbeitsmittel sollte auch im Homeoffice weitestgehend umgesetzt werden [54]. Zur Aufgabenpriorisierung und Arbeitszeitstrukturierung können verschiedene Techniken, wie z. B. die Pomodoro-Technik [12], die ALPEN-Methode [39], das SMART-Prinzip [20], das Eisenhower-Prinzip [21] oder To-do-Listen [12], angewandt werden. Des Weiteren sollten Pausen nicht mit arbeitsrelevanten Themen verbracht, sondern für den sozialen Austausch genutzt oder aktiv gestaltet werden [41, 62]. Pausen sollten Beschäftigte möglichst aktiv gestalten, z. B. durch Bewegung, Dehnübungen, progressive Muskelrelaxation (PMR), Achtsamkeitsübungen [41]. Zur Reduzierung potenzieller Rollenkonflikte kann eine klare räumliche und zeitliche Abgrenzung der Arbeit gegenüber dem Privatleben angestrebt werden [37, 44]. Detachment von der Homeoffice-Tätigkeit kann durch feste Rituale vor und nach der Arbeit gefördert werden [55]. Eine bewusste Aufmerksamkeitslenkung auf positive Alltagsmomente kann als weitere Methode erfolgreiches Detachment fördern [26].

## Fazit für die Praxis

- Die COVID-19-Pandemie stellt seit Anfang des Jahres 2020 eine aktuelle Public-Health-Herausforderung dar, welche auch die Arbeitswelt und ihre Beschäftigten vor anspruchsvolle Aufgaben stellt.
- Für die individuelle Gesundheit der Beschäftigten ist eine Kompetenzerweiterung, z. B. hinsichtlich der ergonomischen Arbeitsplatzgestaltung und Selbstorganisation, begleitet von guter Führungsarbeit, vorteilhaft, um in der neuen Arbeitssituation in der Pandemie gesundheitsfördernd arbeiten zu können.
- Eine entsprechende Organisations- und Führungskultur, welche die gesundheitlichen Bedarfe der Beschäftigten berücksichtigt sowie ihre Arbeitssicherheit und Gesundheit verstärkt in den Vordergrund stellt, ist unabdingbar.
- Betriebliche Abläufe, welche Aufklärung und Edukation miteinbeziehen, bergen großes Potenzial, die Gesundheit der Beschäftigten auch im Homeoffice langfristig und nachhaltig zu fördern und zu erhalten.

## References

[1] Arbeitszeitgesetz (Arbzg) (1994). Arbeitszeitgesetz vom 6. Juni 1994 (BGBl. I S. 1170, 1171), das zuletzt durch Artikel 8 u. Artikel 11 Absatz 2 Satz 2 des Gesetzes vom 27. März 2020 (BGBl. I S. 575) geändert worden ist.

[2] Ausschuss Für Arbeitsstätten (2010). Technische Regeln für Arbeitsstätten: Raumtemperatur (ASR A3.5).

[3] Backhaus N, Brauner C, Tisch A (2019). Auswirkungen verkürzter Ruhezeiten auf Gesundheit und Work-Life-Balance bei Vollzeitbeschäftigten: Ergebnisse der BAuA-Arbeitszeitbefragung 2017. Z Arb Wiss.

[4] Backhaus N, Wöhrmann A, Tisch A (2020). BAuA-Arbeitszeitbefragung: Telearbeit in Deutschland.

[5] Baethge A, Rigotti T (2013). Auswirkung von Arbeitsunterbrechungen und Multitasking auf Leistungsfähigkeit und Gesundheit – Eine Tagebuchstudie bei Gesundheits- und KrankenpflegerInnen.

[6] Beermann B, Amlinger-Chatterjee M, Brenscheidt F (2018). Orts- und zeitflexibles Arbeiten: Gesundheitliche Chancen und Risiken.

[7] Binnewies C, Sonnentag S, Mojza EJ (2009). Feeling recovered and thinking about the good sides of one’s work. J Occup Health Psychol.

[8] Breuer C, Hüffmeier J, Hertel G (2017) Vertrauen per Mausklick: Wie Vertrauen in virtuellen Teams entstehen kann. PERSONALquarterly 2:10–16

[9] Brown R, Duck J, Jimmieson N (2014). E-mail in the workplace: The role of stress appraisals and normative response pressure in the relationship between e-mail stressors and employee strain. Int J Stress Manag.

[10] Brumby DP, Janssen CP, Mark G, Sadowski C, Zimmermann T (2019). How do interruptions affect productivity?. Rethinking productivity in software engineering.

[11] Bundesregierung Telefonschaltkonferenz der Bundeskanzlerin mit den Regierungschefinnen und Regierungschefs der Länder am 15. April 2020. https://www.bundeskanzlerin.de/bkin-de/aktuelles/telefonschaltkonferenz-der-bundeskanzlerin-mit-den-regierungschefinnen-und-regierungschefs-der-laender-am-15-april-2020-1744228. Zugegriffen: 6. Jan. 2021

[12] Cirillo F (2019). The Pomodoro technique. Do more and have fun with time management.

[13] Dainoff M, Maynard W, Robertson M, Salvendy G (2012). Office ergonomics. Handbook of human factors and ergonomics.

[14] Dettmers J, Clauß E, Janneck M, Hoppe A (2018). Arbeitsgestaltungskompetenzen für flexible und selbstgestaltete Arbeitsbedingungen. Gestaltungskompetenzen für gesundes Arbeiten – Arbeitsgestaltung im Zeitalter der Digitalisierung.

[15] Deutsche Gesetzliche Unfallversicherung (2020). Home-Office: So bleibt die Arbeit sicher und gesund.

[16] Deutsche Gesetzliche Unfallversicherung (Dguv) (2019). Bildschirm- und Büroarbeitsplätze. Leitfaden für die Gestaltung.

[17] Deutscher Bundestag (2017). Telearbeit und Mobiles Arbeiten. Voraussetzungen, Merkmale und rechtliche Rahmenbedingungen.

[18] Deutsches Institut für Normung (2000). DIN EN ISO 10075-2:2000 Ergonomische Grundlagen bezüglich psychischer Arbeitsbelastung. Teil 2: Gestaltungsgrundsätze.

[19] Döhner M (2018). Licht am Arbeitsplatz.

[20] Doran GT (1981). There’s a S.M.A.R.T. way to write management’s goals and objectives. Manage Rev.

[21] Eisenhower DD (1954) “Address at the second assembly of the world council of churches, Evanston, Illinois.,” August 19, 1954 (Online by Peters G and Woolley J T, The American Presidency Project)

[22] Empirica (2000). Benchmarking Progress on new ways of working and new forms of business across Europe—ECaTT Final Report.

[23] Fraunhofer-Institut Für Angewandte Informationstechnik Fit (2020). Fraunhofer-Umfrage „Homeoffice“: Ist digitales Arbeiten unsere Zukunft?.

[24] Geiger L, Gerlmaier A, Gerlmaier A, Latniak E (2019). Arbeitsgestaltungskompetenz im Betrieb entwickeln und entfalten: erste Erfahrungen mit dem integrativen Qualifizierungskonzept „SePIAR“. Handbuch psycho-soziale Gestaltung digitaler Produktionsarbeit. Gesundheitsressourcen stärken durch organisationale Gestaltungskompetenz.

[25] Gesetz Über Die Durchführung Von Maßnahmen Des Arbeitsschutzes Zur Verbesserung Der Sicherheit Und Des Gesundheitsschutzes Der Beschäftigten Bei Der Arbeit (Arbeitsschutzgesetz – ArbSchG) (1996) Arbeitsschutzgesetz vom 7. August 1996 (BGBl. I S. 1246), das zuletzt durch Artikel 293 der Verordnung vom 19. Juni 2020 (BGBl. I S. 1328) geändert worden ist

[26] Heckendorf H, Lehr D, Freund H (2016). Wirksamkeit eines internet- und smartphone-basierten Dankbarkeitstrainings zur Förderung der gedanklichen Distanzierung von arbeitsbezogenen Problemen – Sekundäranalyse einer randomisiert-kontrollierten Studie.

[27] Herrmann M, Frey Cordes R (2020). Homeoffice im Zeichen der Pandemie: Neue Perspektiven für Wissenschaft und Praxis?.

[28] International Ergonomics Association Human Factors/Ergonomics (HF/E) Definition and applications. https://iea.cc/what-is-ergonomics/. Zugegriffen: 6. Jan. 2021

[29] Jacobi F (2020). Wie Sie häusliche Isolation und Quarantäne gut überstehen.

[30] Janneck M, Jent S, Weber P (2018). Ergonomics to go: designing the mobile workspace. Int J Human Comput Interact.

[31] Junghanns G (2019) Psychische Belastungen durch digitale Kommunikation – Informationsflut durch elektronische Medien am Arbeitsplatz. Zbl Arbeitsmed 69:119–132. 10.1007/s40664-018-0320-7

[32] Kaluza G (2004). Stressbewältigung. Trainingsmanual zur psychologischen Gesundheitsförderung.

[33] Kappel M (2019). Leitfaden für gesundheitsförderliche Telearbeitsplätze.

[34] Locke EA, Latham GP (1990). A theory of goal setting and task performance.

[35] Lott Y (2017). Selbstorganisiertes Arbeiten als Ressource für Beschäftigte nutzen!.

[36] Macan TH (1994). Time management: test of a process model. J Appl Psychol.

[37] Mann S, Holdsworth L (2003). The psychological impact of teleworking: stress, emotions and health. New Technol Work Employ.

[38] Mark G, Gonzalez VM, Harris J (2005). No task left behind? Examining the nature of fragmented work.

[39] Meier R, Engelmeyer E (2010). Zeitmanagement.

[40] Menz W, Pauls N, Pangert B (2016). Arbeitsbezogene erweiterte Erreichbarkeit: Ursachen, Umgangsstrategien und Bewertung am Beispiel von IT-Beschäftigten. Wirtschaftspsychologie.

[41] Michel A, Wöhrmann AM (2018). Räumliche und zeitliche Entgrenzung der Arbeit: Chancen, Risiken und Beratungsansätze. Psychol Dialog.

[42] Morschhäuser M, Beck D, Lohmann-Haislah A, Bundesanstalt für Arbeitsschutz und Arbeitsmedizin (2014). Psychische Belastung als Gegenstand der Gefährdungsbeurteilung. Gefährdungsbeurteilung psychischer Belastung. Erfahrungen und Empfehlungen.

[43] Müller R (2017). Was ist Arbeitszeit?. Z Bernischen Juristenver.

[44] Mustafa M, Gold M (2013). ‘Chained to my work’? Strategies to manage temporal and physical boundaries among self-employed teleworkers. Hum Resour Manag J.

[45] Nonis SA, Fenner G, Sager JK (2011). Revisiting the relationship between time management and job performance. World J Manag.

[46] Oppolzer A (2010). Gesundheitsmanagement im Betrieb. Integration und Koordination menschengerechter Gestaltung der Arbeit.

[47] Oppolzer A, Badura B, Schröder H, Klose J, Macco K (2010). Psychische Belastungsrisiken aus Sicht der Arbeitswissenschaft und Ansätze für die Prävention. Fehlzeiten-Report 2009. Arbeit und Psyche: Belastungen reduzieren – Wohlbefinden fördern.

[48] Pangert B, Pauls N, Schüpbach H (2016). Die Auswirkungen arbeitsbezogener erweiterter Erreichbarkeit auf Life-Domain-Balance und Gesundheit.

[49] Penners R, Caelers R (2020). Workrave.

[50] Petzold MB, Plag J, Ströhle A (2020) Umgang mit psychischer Belastung bei Gesundheitsfachkräften im Rahmen der Covid-19-Pandemie. Nervenarzt 91:417–421. 10.1007/s00115-020-00905-0 10.1007/s00115-020-00905-0 PMC7100457 32221635

[51] Randolph TH, Oloufa AA (1995). Labor productivity, disruptions, and the ripple effect. Cost Eng.

[52] Richter G, Cernavin O, Klaffke M (2016). Büro als Treiber gesundheitsförderlicher und produktiver Arbeitsbedingungen. Arbeitsplatz der Zukunft: Gestaltungsansätze und Good-Practice-Beispiele.

[53] Richter P, Wegge J, Wittchen H-U, Hoyer J (2011). Occupational Health Psychology – Gegenstand, Modelle, Aufgaben. Klinische Psychologie & Psychotherapie – Ein Lehrbuch.

[54] Schlick C, Bruder R, Luczak H (2018). Arbeitswissenschaft.

[55] Syrek CJ, Weigelt O, Peifer C (2016). Zeigarnik’s sleepless nights: How unfinished tasks at the end of the week impair employee sleep on the weekend through rumination. J Occup Health Psychol.

[56] Tucker P (2003). The impact of rest breaks upon accident risk, fatigue and performance: a review. Work Stress.

[57] Verordnung Über Arbeitsstätten (Arbeitsstättenverordnung – ArbStättV) (2020) Arbeitsstättenverordnung vom 12. August 2004 (BGBl. I S. 2179), die zuletzt durch Artikel 226 der Verordnung vom 19. Juni 2020 (BGBl. I S. 1328) geändert worden ist

[58] Verordnung Über Sicherheit Und Gesundheitsschutz Bei Der Verwendung Von Arbeitsmitteln (Betriebssicherheitsverordnung – BetrSichV) (2015) Betriebssicherheitsverordnung vom 3. Februar 2015 (BGBl. I S. 49), die zuletzt durch Artikel 1 der Verordnungvom 30. April 2019 (BGBl. I S. 554) geändert worden ist

[59] Verwaltungs-Berufsgenossenschaft (Vbg) (2018). Telearbeit: Gesundheit, Gestaltung, Recht.

[60] Warr P (1994). A conceptual framework for the study of work and mental health. Work Stress.

[61] Wendsche J (2014). Wie mache ich Pausen bei der Arbeit richtig? Pausencheck.

[62] Wendsche J, Lohmann-Haislah A (2018). Arbeitspausen gesundheits- und leistungsförderlich gestalten.

[63] Wendsche J, Lohmann-Haislah A (2017). Detachment als Bindeglied zwischen psychischen Arbeitsanforderungen und ermüdungsrelevanten psychischen Beanspruchungsfolgen: Eine Metaanalyse. Z Arb Wiss.

[64] Wood LA (2011). The changing nature of jobs: a meta-analysis examining changes in job characteristics over time.

